# Pathophysiology, Diagnosis, and Management of Coronary Artery Aneurysms: A Review

**DOI:** 10.31083/RCM39702

**Published:** 2025-08-28

**Authors:** Rengin Çetin Güvenç, Abdullah Ayar Al Arfaj, Hola Razouk, Tolga Sinan Güvenç

**Affiliations:** ^1^Department of Cardiology, Okan University School of Medicine, 34959 Istanbul, Turkey; ^2^Department of Cardiology, Istinye University School of Medicine, 34396 Istanbul, Turkey

**Keywords:** coronary arteries, aneurysm, myocardial ischemia, percutaneous coronary intervention, cardiac surgical procedures

## Abstract

Coronary artery aneurysms (CAAs) are frequent entities that are encountered in up to 8% of patients undergoing coronary imaging. The most frequent cause of CAAs is atherosclerotic “positive remodeling” of coronary arteries, while congenital, inflammatory, and traumatic etiologies could also be seen. Aneurysms serve as foci for thrombus formation, which may occlude the aneurysmatic segment or embolize distally. Rupture of an aneurysm is a rare yet potentially catastrophic complication of a CAA. Most aneurysms can be managed medically, while percutaneous exclusion of an aneurysm from coronary circulation is appropriate for CAAs that are prone to rupture or thrombosis. Surgical correction remains the ultimate option for patients who are not amenable to percutaneous management or those with a compelling indication for surgery. This review summarizes the available knowledge on the nomenclature, classification, pathophysiology, diagnosis, and management of CAAs, with a particular emphasis on treatment strategies to mitigate the risks associated with CAAs.

## 1. Introduction

Any major deviation from the normal coronary artery diameter or flow is a matter of great concern, with potential significant implications for long-term outcomes. An ectatic or aneurysmal dilatation is not an uncommon manifestation of coronary artery disease (CAD). Although its true prevalence is unknown as it tends to be asymptomatic, its prevalence has been reported to be 6% in coronary angiography series and 8% in coronary computerized tomography (CT) studies [1, 2]. Due to the nature of this entity, performing randomized controlled studies is difficult if not impossible. As such, case-based approaches and retrospective datasets take center stage in shaping the treatment. This paper aims to explore nomenclature, classification, etiopathogenesis, diagnosis, management strategies and treatment modalities of coronary aneurysms and ectasia in light of current data; considering potential life-threatening consequences such as sudden death, tamponade, heart failure, and myocardial infarction (MI).

## 2. Nomenclature and Classifications

Historically, two terms—namely, coronary artery aneurysm (CAA) and coronary artery ectasia (CAE)—have been used interchangeably to describe dilatations of the coronary arteries, despite having both overlapping and distinct characteristics [3]. According to the prevailing consensus, CAA refers to a localized enlargement of a coronary artery segment, whereas CAE involves a more diffuse dilation affecting more than one-third of the vessel’s entire length. In both cases, the affected segment must have a diameter at least 1.5 times greater than that of an adjacent normal segment. While definitions of CAA and CAE may vary based on factors such as size, affected coronary artery regions, and etiology, one of the most important anatomical distinctions is whether the integrity of coronary artery wall remains intact (i.e., “true” CAA) or not (i.e., “pseudo” CAA) [4, 5, 6, 7].

**Fig. 1.**
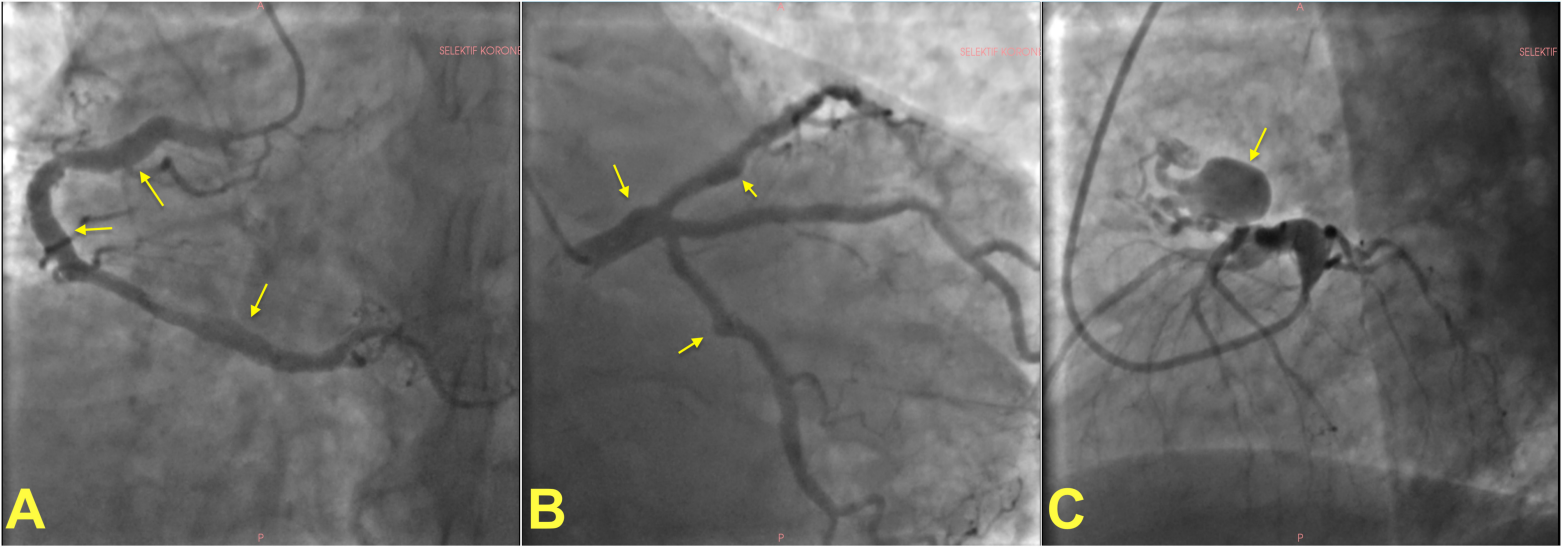
**Invasive angiographic images showing examples of coronary artery ectasia and aneurysms**. (A) shows multiple fusiform atherosclerotic coronary artery aneurysms on the right coronary artery in a patient. Left coronary angiogram of the same patient showing multiple coronary aneurysms on the left main stem, left circumflex and left anterior descending artery (B). A giant saccular aneurysm in a different patient (C). Arrows show aneurysms in all panels.

In true CAA, all three layers of the vessel wall form a sac together. These aneurysms are usually caused by an endogenous mechanism, resulting in either fusiform or saccular dilation [7] (Fig. 1). For saccular aneurysms, the left anterior descending coronary artery is the most common location. Pseudo-aneurysms lack endothelial continuity and generally arise from blunt chest trauma or as a complication of coronary revascularization that can compromise the media and external elastic membrane of the vessel wall [4, 7]. Aneurysms related to percutaneous coronary interventions (PCI) are classified into three subtypes. Aneurysms that grow rapidly during the acute phase (the first 4 weeks) are classified as type 1 and are often accompanied by pericarditis. Aneurysms that develop more slowly during the subacute and chronic phases may remain asymptomatic or cause angina. These aneurysms, which are not clinically aggressive, are classified as type 2. Finally, infective aneurysms with a high risk of fatality are classified as type 3 [8].

Another important consideration is the size of the aneurysm. While some literature defines aneurysms greater than 8 mm as large, others classify those exceeding 20 mm—or in some cases, over 40 mm—as giant CAAs [4, 9, 10]. Giant aneurysms are particularly significant due to their elevated risk of complications, including *in situ* thrombosis, distal embolization, and rupture, necessitating close monitoring even in asymptomatic patients.

Most of the definitions and classifications discussed above primarily apply to localized aneurysms rather than to CAE. For CAE, the classification originally proposed by Markis *et al*. [5], along with a more recent modified version that includes subtypes, is presented in Table 1 [5, 6].

**Table 1.** **Anatomical classification of coronary artery ectasia and aneurysms**.

Original classification of Markis *et al*. [5].	Subtypes proposed by Harikrishnan *et al*. [6].
Type I. Diffuse ectasia in two or three vessels	Type Ia. Diffuse in three vessels
	Type Ib. Diffuse in two vessels, localized (discrete) in one vessel
	Type Ic. Diffuse in two vessels
Type II. Diffuse ectasia in one vessel localized disease in another vessel	Type IIa. Localized ectasia in one vessel only
	Type IIb. Localized ectasia in two vessels
Type III. Diffuse ectasia in one vessel	No subtypes
Type IV. Localized ectasia	Type IVa. One vessel involved
	Type IVb. Two vessels involved
	Type IVc. Three vessels involved

Classification schemes proposed by Markis *et al*. [5] and Harikrishnan *et al*. [6].

## 3. Etiopathogenesis

Numerous studies have identified various predisposing and/or associated factors related to CAAs, but the precise cause of vascular damage in the development of these aneurysms remains unclear. Several factors contribute to CAA formation, and atherosclerosis is recognized as the primary underlying cause, accounting for 52% of cases [1]. However, it is important to note that only around 1.5% of patients with atherosclerotic disease will go on to develop aneurysms [11, 12]. The focus is often placed on the inflammatory process, which typically involves multiple coronary arteries and is triggered by lipid accumulation beneath the endothelial layer, particularly in the presence of risk factors that promote atherosclerosis. This process results in the degradation of extracellular matrix proteins [12]. At least one study suggests that excessive angiotensin-converting enzyme activity may initiate the inflammatory response, leading to damage to both the internal and external vessel membranes [13]. In contrast, inflammatory diseases such as Kawasaki disease result in a vasculitic process that compromises the structural integrity of the arterial walls. In later stages, severe calcification in the vessel wall and circumferential luminal narrowing may accompany aneurysmal and/or ectatic segments [14, 15].

Additionally, congenital factors can contribute to CAA, although these remain poorly understood. Genetic mutations, abnormal blood flow dynamics, and environmental influences during development are believed to play a role. In some cases, congenital coronary aneurysms have been observed alongside renal and carotid aneurysms in individuals with fibromuscular dysplasia [16, 17]. The relationship between these factors in the development of aneurysms is complex. Aneurysmal changes due to atherosclerosis or inflammation may also be linked to a genetic predisposition. Over time, atherosclerosis can develop within an aneurysmal dilation influenced by genetic factors, and disturbances in blood flow may lead to endothelial damage, potentially initiating the progression of atherosclerosis.

Most CAAs affect right coronary (40%) and left anterior descending (30%) arteries, while circumflex and left main coronary arteries are seldom involved. In patients who have undergone coronary artery bypass grafting (CABG), rare but potentially dangerous aneurysms can occur in the saphenous vein grafts [4, 7].

Direct trauma to the coronary arteries, particularly related to PCI or surgical revascularization, constitutes an important fraction of CAAs. During stent implantation, factors such as edge dissection, high inflation pressures, oversized stents, and stent fractures can contribute to the development of a CAA [8]. Different mechanisms were proposed for CAAs related to bare metal stents (BMS), drug-eluting stents (DES) and drug-coated balloons (DCB) [18]. In the case of BMS, local pressure is the primary factor, while inflammatory and allergic reactions are often implicated [19]. For DES and DCB, in addition to procedural effects and hypersensitivity reactions, it is proposed that drug release helps prevent stenosis in the long term, but also delays endothelialization and weakens the arterial wall, resulting in CAAs [8, 18, 20].

Pivotal DES and BMS comparison studies showed no difference in overall prevalence in the short-term follow-up, and the absolute incidence of CAAs was relatively small with both types of stents (1.1% with DES and 0.8% with BMS) [8, 21]. A recent retrospective study suggested that only 0.8% of interventions involving DCB were complicated by the development of a CAA [22], although others have suggested that the rate of this complication could be much higher in patients undergoing DCB for a chronic total occlusion [23]. In a noteworthy case, Huang *et al*. [18] observed multiple aneurysms 6 months after an intervention for in-stent stenosis with DCB. While there is growing evidence supporting the development of aneurysms, the rate of poor outcomes in these cases remains low. This effect may be attributed to the drug release, which helps reduce restenosis and major adverse cardiac event (MACE) rates but also hampers the healing process after the initial vascular injury caused by balloon inflation.

## 4. Clinical Presentation and Diagnosis

Symptoms at presentation can vary widely, ranging from asymptomatic cases to sudden death. However, most cases are asymptomatic and can be incidentally detected during coronary angiography or a coronary CT scan [24]. Factors increasing myocardial oxygen demand, such as pregnancy or severe infection, may cause development of symptoms in a previously asymptomatic patient. After a triggering event, thrombosis of the aneurysm (with or without peripheral embolization of the thrombus) or rupture of an aneurysm may lead to catastrophic complications such as MI, arrhythmia, sudden death, or cardiac tamponade [25, 26, 27, 28]. Similar to type 2 aneurysms seen after stent implantation, patients with Kawasaki disease can present with pericarditis and mild pericardial effusions [8, 15].

The migration of thrombus from the aneurysm to the distal portion of the parent vessel, or decreased blood flow within the aneurysm or ectatic vessel, can result in acute coronary syndrome, arrhythmias and sudden death [29, 30]. In cases involving coronary artery fistulas, high-output heart failure occurs as a result of the increased volume load caused by the aneurysm or coronary-cameral fistulas [25]. MI or arrhythmias related to CAAs may also lead a reduced left ventricular systolic function with subsequent low-output heart failure.

When symptoms occur after an inciting event, patients may experience chest pain, exertional angina, dyspnea, and arrhythmias. The size of the aneurysm, along with co-existing CAD and associated risk factors, may influence the nature of the symptoms, their presentation, and potential complications. Although infrequent, aneurysms might rupture, causing tamponade and sudden death. Exercise-induced dyspnea and chest pain during physical activity may occur due to the slow flow caused by ectatic vessels [25, 26, 27, 28, 29, 30].

### Diagnosis of Coronary Artery Aneurysms

Non-invasive tools, including echocardiography, CT, and magnetic resonance imaging (MRI), can be used to detect CAAs. Nevertheless, coronary angiography is considered the best method for the assessment of coronary anatomy due to its high spatial and temporal resolution [7]. Recent utilization of intravascular ultrasound (IVUS) imaging has provided valuable insights for diagnosis and classification of CAAs. IVUS offers detailed insights into the arterial wall and lumen, aiding in the differentiation between pseudo-aneurysms and true aneurysms, as well as clarifying the presence or absence of atherosclerosis [31, 32].

Echocardiography can be utilized to provide information on etiology and also to evaluate complications accompanying aneurysm, such as heart failure or pericardial effusion [25]. Incidentally, giant aneurysms can be visualized during an echocardiographic examination, as well as in CT and MRI scans of asymptomatic patients undergoing these modalities for other reasons [7, 25, 33].

The size of the aneurysm, the presence of thrombus, and the degree of calcification can be more accurately assessed by CT angiography [34]. CT could also be used to evaluate the distribution, maximum diameter, presence of intraluminal thrombus, number and extent of CAAs, and associated complications including MI [34, 35].

## 5. Management

The ideal treatment approach for managing CAAs remains debated, even when focusing solely on complications related to CAD, due to the clinical and anatomical variability as well as the often asymptomatic nature of the condition. Strategies to manage CAAs include medical treatment, PCI, or surgery, particularly in the presence of complicated CAAs. According to the findings from the international Coronary Artery Aneurysm Registry, the stenotic segment was primarily treated percutaneously (53%), with the majority of patients undergoing some form of revascularization procedure (69%) [36]. However, due to the absence of randomized clinical trials, most available data consists of case-based approaches and personalized treatment strategies.

### 5.1 Medical Treatment 

The goal of treatment for CAA is to modify risk factors and prevent acute complications and/or long-term MACE. Antiplatelet and anticoagulant treatments play a key role to prevent thrombus formation within aneurysms and distal embolization, which may lead to acute coronary events or life-threatening arrhythmias. Additionally, CAD guidelines recommend using antiplatelet agents for conditions involving atherosclerosis, slow blood flow, and ischemia associated with ectatic coronary arteries [37, 38]. A major area of controversy concerns asymptomatic patients and anticoagulant treatment, except in the case of Kawasaki disease, due to the lack of high-quality supporting data [15]. A study conducted on asymptomatic patients showed a high frequency of major adverse outcomes during the 5-year follow-up [39]. As such, close monitoring and adjustment of coronary risk factors were recommended. Administration of intravenous immunoglobulin during the acute phase of Kawasaki disease decreased the frequency of CAAs by 8%. Japanese guidelines recommend anticoagulant treatment in patients with Kawasaki disease who have particularly large and recurrent aneurysms [15, 40].

Although previous small-scale retrospective studies reported no difference in major adverse outcomes between anti-platelet and anticoagulant use for CAA, an observational study suggested the usefulness of anticoagulation, especially in the presence of acute MI, with no major adverse events among patients receiving effective anticoagulant therapy [29, 41, 42].

### 5.2 Coronary Revascularization

In ST-elevation MI, restoring coronary flow is the top priority, and every effort is made to achieve this as quickly as possible. Given the inherent delays associated with CABG, primary PCI is considered the most effective method of revascularization in this situation. Other patients that benefit from revascularization are those with non-ST elevation MI, unstable angina pectoris, stable angina that is refractory to medical treatment, and high-risk coronary anatomy such as multi-vessel disease or left main coronary stenosis [37, 38].

#### 5.2.1 Percutaneous Coronary Intervention

A variety of PCI techniques, such as balloon angioplasty, DCB, BMS, DES, coil embolization and stent grafting, have been employed for the treatment of CAA and CAE. Two methods, stent-assisted coil embolization and stent grafting, stand out for their ability to occlude aneurysms and restore flow [4, 7].

**Fig. 2.**
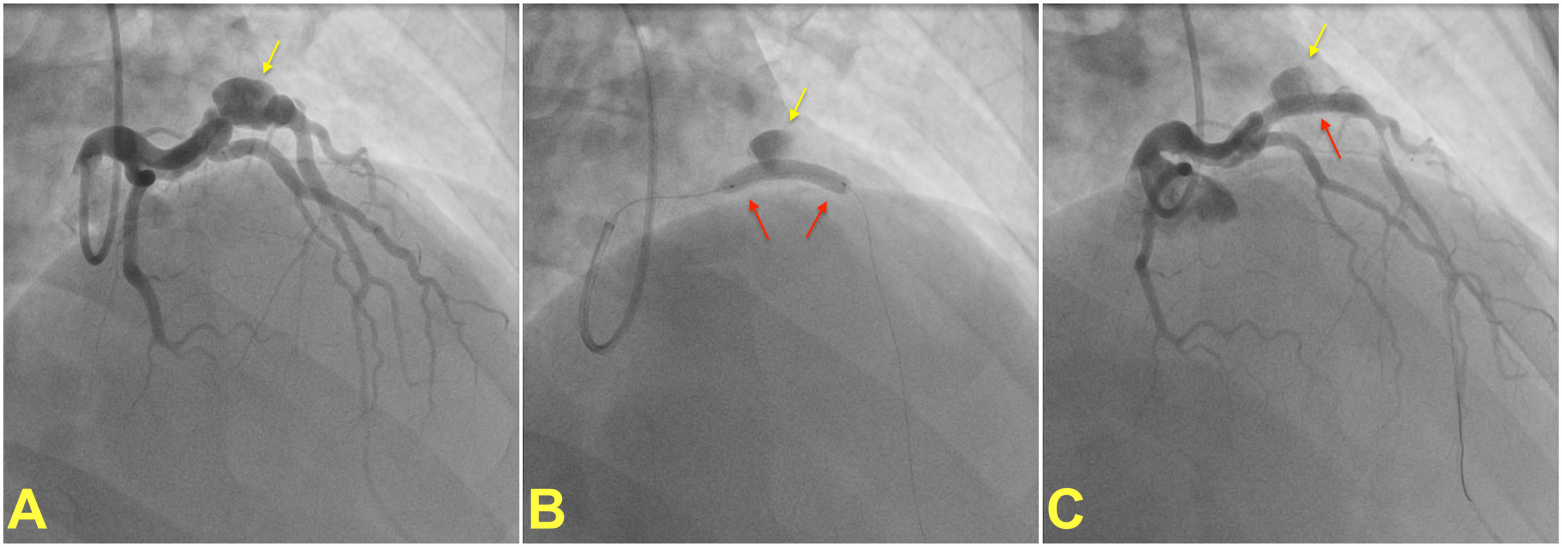
**Percutaneous treatment of a coronary artery aneurysm**. (A) shows coronary angiogram of a patient with a saccular coronary aneurysm (yellow arrow) on the left anterior descending artery. A covered stent (red arrow) is placed and deployed to isolate the aneurysm from the coronary circulation (B). (C) shows the final result after post-dilatation of the covered stent with a noncompliant balloon. Note that the contrast within the aneurysm is residual and does not indicate stent failure. Images courtesy of Dr. Muslum Sahin.

The aim of combining a stent with coil embolization is to maintain the patency of the flow within the main branch while simultaneously sealing off the aneurysm. It is mostly preferred when the CAA involves a major side branch artery, for bifurcation lesions and wide-neck aneurysms [43, 44, 45]. Stent graft is an alternative technique and is often preferred to seal relatively small saccular aneurysms when the coronary anatomy is suitable [46, 47] (Fig. 2). However, due to their stiff nature, stent grafts are not ideal for tortuous or severely calcified diseased vessels, as they may pose a risk of coronary dissection. Other concerns associated with covered stents include the closure of nearby side branches close to the aneurysm site, stent thrombosis, and the recurrence of restenosis [4, 48].

On the other hand, in cases of large aneurysms (greater than 20 mm) or those located at critical locations, such as the left main stem or saphenous grafts, procedures done with either method carry a higher risk of failure. Procedural failure includes aneurysm leakage, which may necessitate additional coil embolization or the placement of more stents. Such situations could increase thrombus burden and negatively impact the procedure’s success [47, 49, 50, 51].

Other important problems that may arise during the intervention include diameter mismatches, sizing and positioning difficulties, thrombus accumulation within the aneurysm and its neck and embolization of thrombi within the vessel wall, particularly in Kawasaki patients [15, 52]. Diameter mismatch may lead to additional complications like stent migration and distal embolization, resulting in recurrent MI and death. Due to flow stagnation and increased risk of thrombus formation, administration of glycoprotein IIb/IIIa inhibitors and treatment with other anticoagulants can be considered in such cases. In a case report, the operators managed to restore flow using a novel stent retriever device in a patient with intracoronary thrombosis within a CAA that was unresponsive to conventional percutaneous thrombectomy and intracoronary thrombolytics [53].

Studies have shown that aneurysmal or ectatic culprit vessels carry low angiographic success rates compared to non-aneurysmal culprit lesions [54, 55]. This increases the risk of stent thrombosis, MI, and both short- and long-term mortality. A study by Iannopollo *et al*. [55] found that aneurysmal culprit lesions were independently associated with all-cause mortality, recurrent MI, and a 16.5% increased relative risk for stent thrombosis, with the majority of these events occurring within the first month after MI. The use of optical coherence tomography and IVUS is recommended to help minimize complications in treating CAA and CEA. These technologies assist in clarifying coronary anatomy during the initial procedure, guiding stent placement, identifying stent fracture sites in subsequent procedures, and assessing stent malapposition [18, 56]. As aforementioned, available evidence does not suggest an increased risk for *de novo* aneurysms in patients treated with DCB and DES [21, 22]. In patients undergoing PCI for a coronary segment with a CAA, findings from the CAA registry suggest that BMS (as compared to DES) was associated with a higher incidence of MACE and death [36]. Novel stent designs, such as self-apposing stents and micro mesh stents, as well as routine use of intravascular imaging in patients with CAAs may further reduce the risk of periprocedural and long-term complications [57].

Despite the factors leading to unfavorable outcomes—such as diameter mismatches, difficulties in selecting the appropriate stent size, thrombus shift and burden, and elevated no-reflow rates—as noted earlier, PCI techniques remain the leading treatment choice, especially for acute MI. Adherence to interventional guidelines during the acute phase of coronary syndromes, as well as throughout the subsequent follow-up period, constitutes a rational therapeutic strategy for addressing CAA and CAE [37, 38]. Nevertheless, the approach remains controversial in asymptomatic patients. In cases where the aneurysmal segment is large and might pose a risk of rupture, as well as when a cardiac surgery or interventional procedure is planned for another reason, interventional treatment may be considered. An additional indication might be progression of aneurysmal dilatation given that this may increase the tendency for rupture of the CAA [58].

#### 5.2.2 Surgical Revascularization

There are no randomized studies comparing CABG versus PCI to determine the best option for treating CAA and CAE. All available data in the literature comparing CABG and PCI are based on atherosclerotic coronary disease [37, 38]. As a result, either projections from studies not involving CAA or expert opinion are used to guide indications for surgery for CAA.

According to available guidelines, CABG is the preferred approach in patients with left main coronary artery (LMCA) and LMCA-equivalent lesions and those with multi-vessel disease, particularly if not all of the lesions were amenable to treatment with PCI [38]. Patients with diabetes and those with heart failure at baseline are also candidates for CABG given the proven superiority of CABG over PCI in these scenarios [59]. Patients who are not ideal candidates for PCI, such as those with giant aneurysms where the risk of rupture is high or those with significant diameter mismatch where stent apposition to vessel wall would be suboptimal, may constitute additional indications for CABG [1, 14, 60].

In Kawasaki disease, the success of PCI procedures is limited for both single-vessel and multivessel focal aneurysms [15]. At least two studies indicated superior long-term outcomes with CABG over PCI [14, 61]. A third study found no difference between CABG and PCI in the primary endpoint (ST elevation MI and all-cause mortality), although the need for repeat revascularization was also higher in PCI group in this latter study [62]. Most patients included in these studies were children or younger adults who had a lower risk of CABG-related complications, which might have contributed to the overall benefit observed with CABG [61]. Thus, it remains uncertain whether this superiority of CABG over PCI extends to those in whom older age and comorbidities may offset the benefit observed with CABG.

Another concern is type 3 infected aneurysms that develop after stent implantation. In this relatively rare situation, surgery should be the first choice due to the significantly increased risk of mortality [8]. Surgical intervention is typically preferred in emergencies such as rupture or tamponade, although interventional methods may be an option for patients deemed as high-risk for CABG [4, 7]. Interventional closure of fistulas using vascular plugs or covered stents remains an alternative to CABG in those with coexisting coronary-cameral fistulas [63].

In summary, based on studies comparing CABG and PCI in patients with CAD, surgery is preferred for patients with CAAs at high risk for complications with PCI, such as left main or multi-vessel disease, regardless of the presence of an aneurysm. Surgery is also indicated in cases where multiple surgical interventions are required, such as valve surgery; in situations where PCI methods cannot reduce risk, such as large or multiple aneurysms in diffuse disease; or where the procedural risk is likely to cause more harm than benefit, such as in cases with a high risk of rupture or tamponade.

## 6. Conclusions

Coronary artery aneurysms are encountered in a significant proportion of patients undergoing CT or invasive angiography. In most patients, CAAs and CAEs are associated with atherosclerotic CAD, while traumatic, iatrogenic and congenital CAAs are encountered less frequently. In addition to increasing the risk of coronary thrombosis due to flow stagnation, a rare but catastrophic consequence of CAA is rupture with subsequent tamponade and death. Smaller aneurysms can be managed medically, while revascularization should be considered in patients with an acute coronary event or those in whom a large portion of myocardium is jeopardized due to concomitant obstructive CAD. For asymptomatic patients, the chief concern is the risk of rupture and thrombosis within the aneurysmal sac that may cause an acute coronary event, and the decision to proceed with revascularization should be individualized. While most CAAs are amenable to revascularization with PCI, CABG is an option for those with a coexisting indication for surgery or those with Kawasaki disease.
